# Cross-sectional survey of gender differences in gross motor skills among preschool children in Jinhua City, China

**DOI:** 10.1016/j.heliyon.2024.e39872

**Published:** 2024-10-26

**Authors:** Biqing Chen, Yubo Liu, Jingwei Tang, Jing Wang, Feng Hong, Weibing Ye

**Affiliations:** a Department of Sports Operation and Management, Jinhua University of Vocational Technology, Jinhua, Zhejiang Province, China; b Institute of Human Movement and Sports Engineering, College of Physical Education and Health Sciences, Zhejiang Normal University, Jinhua, Zhejiang Province, China; c Qixin College, Ningbo Tech University, Ningbo, Zhejiang Province, China; d Institute of Culture Creativity, Weifang Vocational College, Shandong Province, China

**Keywords:** Fundamental motor skills, Locomotor, Object control, Ball skill, Gender differences, Preschool

## Abstract

**Background:**

While gender differences in ball skills are widely recognized, the differences in locomotor skills remain unclear. This study aims to explore these gender differences among preschool children in Jinhua City, China, and to examine the influence of evolutionary factors and local cultural activities on these skills.

**Methods:**

A cluster random sampling method was employed to select 777 preschool children aged 5.1–6.7 years from seven kindergartens in Jinhua, Zhejiang Province, Eastern China. The Test of Gross Motor Development-Third Edition (TGMD-3) was used to assess GMS, including locomotor and ball skills.

**Results:**

Boys outperformed girls in ball skills, such as underhand throwing, catching, two-handed striking, and kicking. However, no significant overall gender differences were found in locomotor skills. A deeper analysis revealed that boys excelled in running-related tasks, whereas girls performed better in skipping-related tasks. These findings suggest that TGMD-3 locomotor skills should be divided into running-related and jumping-related categories. The results also show that not all ball skills are dominated by boys, indicating the impact of environmental factors. The findings highlight the role of both evolutionary factors and local cultural activities in shaping these specific gender differences.

**Conclusion:**

The study's cross-sectional design provides a snapshot of existing gender differences in motor skills among preschool children. The results suggest the need for gender-sensitive physical education programs to address these disparities early on. Future research should utilize longitudinal methods and objective measures to further understand the development of these skills over time and the underlying factors contributing to these differences.

## Highlights

• Boys excel in running; girls excel in skipping.
• No gender difference in overhand throwing, one-hand strike, and forehand strike.
• Cultural and evolutionary factors influence motor skill development.

## 1 Introduction

### 1.1 Importance of physical activity and gross motor skills

Engaging in physical activity is essential for health and well-being across the lifespan, offering numerous documented benefits[1,2]. A global survey involving 163 countries reported that the age-standardized prevalence of physical inactivity was 31.3 % in 2022, with an increasing trend, and higher inactivity rates among females compared to males [3]. However, physical activity levels among children and adolescents remain seriously low a global scale [4], particularly among females [5]. This disparity is evident from early childhood, where females demonstrate lower levels of moderate-to-vigorous physical activity (MVPA) compared to males [6].

Gross Motor Skills (GMS), also referred to as Fundamental Motor Skills (FMS), include essential learned movement patterns such as locomotor skills (e.g., running, jumping) and ball skills (e.g., kicking, catching) that are essential for engaging in more complex physical and sporting activities [7]. These skills not only enhance MVPA [8], but also improve cardiorespiratory fitness[9,10], and reduce the risk of obesity in children by promoting a more active lifestyle [11]. Furthermore, GMS contribute significantly to cognitive and academic development [12], influencing physical activity levels throughout an individual's lifespan [13]. Longitudinal research indicates that GMS can predict changes in Body Mass Index (BMI), serving as a more sensitive measure than general physical activity or fitness metrics [14]. Additionally, motor skills are significant predictors of school performance in the short term [6]. These findings highlight the necessity for effective interventions focusing on GMS to promote long-term health and academic benefits.

### 1.2 Gender differences in gross motor skills

The complexity of gender differences in GMS is highlighted by various studies, each presenting different findings. For instance, our previous systematic review and meta-analysis indicated that boys generally showed higher proficiency in global GMS and object control skills compared to girls, while differences in locomotor skills were less pronounced but tended to favor girls [7],Similar trends were observed in our empirical studies [15,16]. Spessato et al. [17]found that boys excel in running and galloping, whereas Pang et al. [18]reported that girls overcome boys in both locomotor and object control skills. Conversely, Robinson et al. [19] found results that entirely contradict Pang et al.'s findings, with boys outperforming girls in both locomotor and object control raw scores. Additionally, a study involving 712 five-year-olds using the Bruininks–Oseretsky Test, Second Edition, revealed that females outperformed males in most motor skill tasks, with statistically significant gender differences observed in eight out of 14 tests [20]. These discrepancies underscore the need to monitor gender differences in motor skills across various ethnic, regional, and cultural backgrounds, especially in a multi-ethnic country like China where several cultural factors may influence motor skill development [21,22]. Understanding these differences is crucial for developing tailored interventions that address the unique needs of boys and girls in diverse contexts.

### 1.3 Educational transition and its impact on motor Skill development

The transition from preschool to primary school marks a pivotal phase for children in China, particularly when they begin formal schooling, which includes structured physical education programs, standardized physical fitness assessments, and more systematic sports activities. This period is also critical for the development of motor skills as children's physical activity patterns can significantly change [22]. In Chinese preschools, children's abilities, especially in physical activities, are assessed without quantitative grades, allowing for a more natural expression of motor skills. However, upon entering primary school, the educational dynamics change significantly, as academic subjects and physical education are formally graded. This introduction of grading can recall the Matthew effect [23], where early advantages in motor skills could amplify, while initial disadvantages may worsen. Additionally, the expectations set by teachers and parents, known as the Pygmalion effect [24], and phenomena such as learned helplessness, can significantly influence children's performance, leading them to meet or even exceed those expectations. These dynamics highlight the need for structured support during this critical educational transition to ensure equitable development among all children, particularly in their physical education.

### 1.4 Purpose of the study

This study employs the third edition of the Test of Gross Motor Development (TGMD-3), revised by Dr. Ulrich [25], to assess the GMS of preschool children in the urban areas of Jinhua. By analyzing gender differences in these skills, the research aims to deepen the understanding of disparities between boys and girls and explore the specific sub-skills within the TGMD-3 that contribute to these differences. Additionally, this study seeks to identify the evolutionary and cultural factors influencing these gender differences, thereby informing the development of physical education curricula that prioritize early engagement and targeted skill acquisition. The findings will support the comprehensive growth of gross motor skills in children across China and provide insights into child development within diverse cultural contexts.

## 2 Materials & methods

### 2.1 Participants

**Fig. 1:**
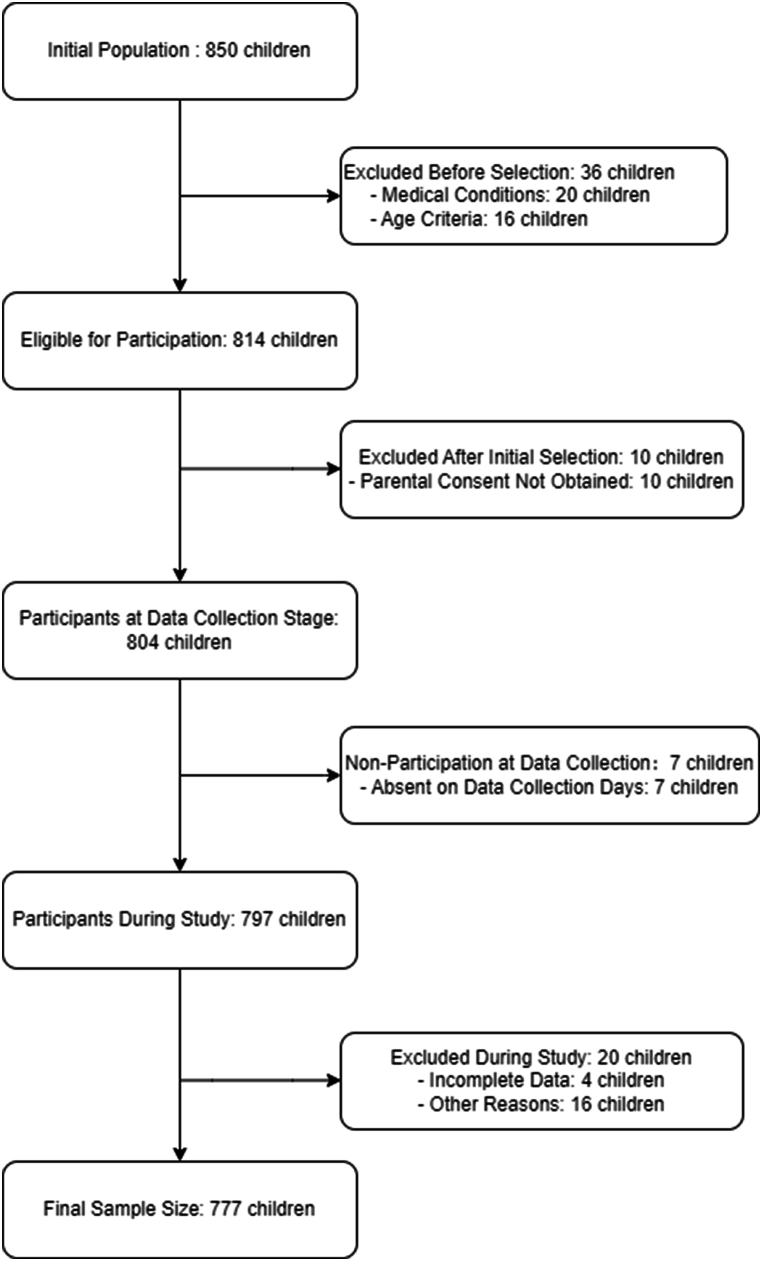
Flowchart of Participant Selection and Exclusion Process.

This study employed a cluster random sampling method to select 850 children from seven kindergartens in the urban area of Jinhua, Zhejiang Province, Eastern China, a city with a population of approximately 1 million. The children selected for this study were all preparing to enter primary school. The inclusion criteria required participants to be in preschool, as some parents choose to delay their child's entry into primary school, resulting in some children being older than 6 years. Children were excluded from the study if they had developmental disorders (e.g., cerebral palsy, epilepsy, Down syndrome, autism spectrum disorders, attention deficit hyperactivity disorder), severe organ diseases which do not allow to practice physical activities (e.g., heart disease, asthma), significant physical deformities (e.g., scoliosis, polydactyly, foot deformities, bowlegs), or if they had suffered physical injuries within the last six months that had not fully healed. Children over 7 years old and those with incomplete data on major test items were excluded. After applying these criteria, 37 children were excluded from the study, leaving 777 children for analysis. Details of the selection and exclusion process are provided in Fig. 1.

### 2.2 The Test of Gross Motor Development (TGMD)

The Test of Gross Motor Development (TGMD) is a process-oriented assessment for children aged 3–10 years that evaluates large muscle motor skills. It was revised in 2000 to the second edition (TGMD-2) and further modified by Dr. Ulrich [25]. The TGMD-3 scoring sheets became available in 2015 and have been validated for reliability and validity in multiple countries [26,27]. Chinese scholar Diao Yucui et al. [28] also confirmed the reliability and validity of the TGMD-3, with Cronbach's alpha coefficients ranging from 0.76 to 0.84 across different scales and total score. The retest reliability Pearson correlation coefficients for the total scale and sub-scales were above 0.93, demonstrating good reliability and validity for assessing the gross motor skills of Chinese children aged 3–10 years [28].

The TGMD-3 includes two skill categories: six locomotor skills (LS) — running, galloping, sliding, skipping, horizontal jumping, and hopping — and seven ball skills (BS) — overhand throwing, underhand throwing, two-hand catching, one-hand stationary striking, forehand striking of the self-bounced ball, two-hand striking a stationary ball, and kicking a stationary ball. For the test evaluation, each skill was categorized into 3–5 components, with each component scored as either 1 (present) or 0 (absent). The maximum possible score was 46 points for LM and 54 points for BS, with a total possible score of 100.

The children's GMS competence test was conducted in the indoor gymnasium of each kindergarten after obtaining permission from the administrators. Each skill was demonstrated once by the examiners, followed by two attempts by the child. Both attempts were scored, and the scores were summed to obtain the total score for each skill. Two motor skill subset scores (LM and BS) were computed from the sum of raw scores from each subset, and the total gross motor skills score was the sum of LM and BS. To ensure consistency and accuracy, this study followed a strict testing protocol conducted by two professionally trained examiners. The testing equipment included child-specific gear such as tennis balls, mini basketballs, soccer balls, fixed ball stands, baseballs, measuring tapes, soccer markers, recording sheets, video cameras, and laptops.

### 2.3 Additional assessment components

The basic characteristics of the children, including body weight (kg) and height (m), were measured using a weight and height scale, accurate to the nearest 0.1 kg and 0.1 cm, respectively. BMI was then calculated using the formula [weight (kg)/height (m)^2^]. These measurements were conducted in the empty hall of each kindergarten during school hours. Additionally, demographic data (age and gender) provided by the teachers were recorded for analysis. This study took several measures to minimize potential sources of bias. Selection bias was addressed by employing a cluster random sampling method to select participants from various kindergartens, ensuring a representative sample. Information bias was minimized by using standardized instruments and protocols, such as the Test of Gross Motor Development-3 (TGMD-3), for assessing motor skills. Additionally, all data collectors received rigorous training to ensure consistency and accuracy in data recording.

Before the study, comprehensive information regarding the research was provided to principals, teachers, guardians, and participating children through written documents, formal letters, and oral briefings. Informed consent was obtained from all guardians via signed consent forms. The study design and all associated assessment procedures received ethical approval from the Institutional Ethical Commit-tee of Zhejiang Normal University, under approval number ZSDR2019013. This study adhered to the principles of the Helsinki Declaration. The testing period was from March to August 2021, during which the kindergartens were not affected by COVID-19 lockdowns.

### 2.4 Statistical methods

Statistical analyses were performed using JASP (Version 0.19.0) [JASP Team, 2024]. Descriptive statistics, including mean and standard deviation (Means ± SD), were used to characterize the demographic features of the participants. The Shapiro-Wilk test was used to assess the normality of the data, and Levene's test was used to evaluate the equality of variances. Due to the small p-values in many of our normality and homogeneity of variances tests, Welch's *t*-test was primarily used to assess gender differences. Additionally, effect sizes were calculated using Cohen's d, with values interpreted as small (0.2), medium (0.5), and large (0.8) effects, reflecting the nature of our data and analysis methods. These thresholds align with Cohen's guidelines and are consistent with the practical significance observed in similar educational and developmental research. The significance level was set at p < 0.05.

## 3 Results

### 3.1 Demographic characteristics of the study participants

The demographic characteristics of the study participants are summarized in Table 1. Significant gender differences were identified in height and weight among preschool children, with boys generally being taller and heavier than girls. The effect sizes for these differences were small to medium (Cohen's d = 0.225 for height and 0.201 for weight), indicating a modest but meaningful difference between boys and girls. No significant differences were found in age and BMI between genders, with very small effect sizes, suggesting negligible differences in these parameters.

**Table 1:** Basic Information of preschool children (Means ± SD).

Description	All(n = 777)	Boys(n = 395)	Girls(n = 382)	t	*p*	Cohen's d
Age (years)	5.9**1** ± 0.4**7**	5.88 ± 0.48	5.94 ± 0.46	−1.807	0.071	−0.130
Height (cm)	121.66 ± 6.16	122.34 ± 6.51^a^	120.96 ± 5.70^a^	3.143	0.002	0.225
Weight (kg)	21.85 ± 3.92	22.24 ± 3.93^a^	21.45 ± 3.87^a^	2.799	0.005	0.201
BMI (kg/m^2^)	14.69 ± 1.70.	14.77 ± 1.58	14.60 ± 1.81	1.417	0.157	0.102

a The *t*-test refers to a two-tailed independent sample test comparing boys and girls, Statistical significance is denoted by p < 0.05.

### 3.2 Gender differences in total and sub-scale scores of TGMD-3 among preschool children

Table 2 summarizes the gender differences in total GMS scores and sub-scales. The study found that boys scored significantly higher than girls in ball skills, with a small to medium effect size (Cohen's d = 0.322). For the total GMS score, boys also scored higher, but the effect size was just below the threshold for small effects (Cohen's d = 0.189), indicating a negligible to small difference. No significant gender difference was observed in locomotor skills scores, with a very small effect size (Cohen's d = −0.029), suggesting negligible differences between boys and girls in this subset skill.

**Table 2:** Gender differences in total GMS score and sub-scales (Means ± SD).

Description	Boys(n = 395)	Girls(n = 382)	t	*p*	Cohen's d
Locomotor Skills Score	30.89 ± 6.48	31.06 ± 5.70	−0.398	0.691	−0.029
Ball Skills Score	29.61 ± 7.81^a^	27.30 ± 6.47^a^	4.493	<0.001	0.322
Total GMS Score	60.50 ± 12.19^a^	58.36 ± 10.32^a^	2.637	<0.009	0.189

a The *t*-test refers to a two-tailed independent sample test comparing boys and girls, Statistical significance is denoted by p < 0.05.

### 3.3 Gender differences in specific locomotor skills

**Fig. 2:**
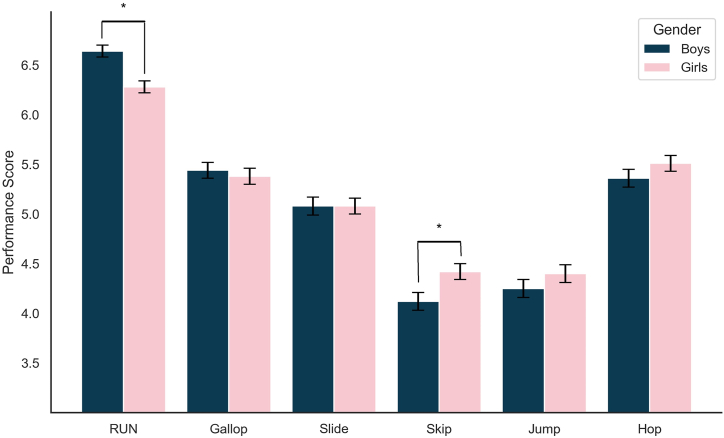
Performance Scores of Boys and Girls in Various Locomotor Skills. * Statistical significance between boys and girls is indicated (p < 0.05).

Although Table 2 shows no significant gender differences in the overall locomotor skills of preschool children, Table 3 provides a detailed breakdown of the locomotor skills scores for boys and girls for each skill. Boys scored significantly higher in running (p < 0.05), with a small to medium effect size (Cohen's d = 0.287). In contrast, girls outperformed boys in skipping (p < 0.05), but the effect size was negligible (Cohen's d = −0.187), indicating that the difference in performance was not practically significant. No significant gender differences were observed in galloping, sliding, jumping, or hopping skills (p > 0.05), with minimal effect sizes (Cohen's d ranging from <0.001 to −0.090). Fig. 2 illustrates these differences in detail across various locomotor skills.

**Table 3:** Detailed scores of locomotor skills by gender in preschool children (Means ± SD).

Description	Boys(n = 395)	Girls(n = 382)	t	*p*	Cohen's d
Run Score	6.64 ± 1.25^a^	6.291 ± 1.22^a^	3.995	<0.001	0.287
Gallop Score	5.44 ± 1.68	5.38 ± 1.58	0.543	0.587	0.039
Slide Score	5.08 ± 1.81	5.08 ± 1.65	<0.001	1.000	<0.001
Skip Score	4.12 ± 1.69^a^	4.42 ± 1.51^a^	−2.603	0.009	−0.187
Jump Score	4.25 ± 1.74	4.40 ± 1.75	−1.159	0.247	−0.083
Hop Score	5.36 ± 1.73	5.51 ± 1.50	−1.255	0.210	−0.090

a The *t*-test refers to a two-tailed independent sample test comparing boys and girls, Statistical significance is denoted by p < 0.05.

### 3.4 Gender differences in specific ball skills

**Fig. 3:**
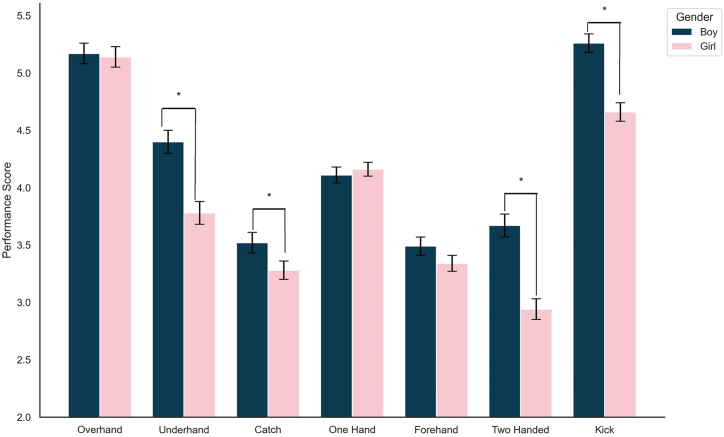
Performance Scores of Boys and Girls in Various ball Skills 1

Although Table 2 shows that boys generally outperform girls in ball skills, Table 4 provides a detailed comparison of these skills between boys and girls among preschool children, using mean and standard deviation scores. The analysis reveals significant gender differences in four specific skills, as indicated by p-values less than 0.05. However, from the perspective of effect sizes, only three skills—underhand throwing, two-handed striking, and kicking, showed small to medium effect sizes (Cohen's d ranging from 0.310 to 0.408), suggesting meaningful differences. The catching skill, despite showing a significant p-value, had an effect size below the threshold for a small effect. Additionally, these differences are visually represented in Fig. 3, which illustrates the gender variations across different ball skills.

**Table 4:** Detailed scores of ball skills by gender in preschool children (Means ± SD).

Description	Boys(n = 395)	Girls(n = 382)	t	*p*	Cohen's d
Overhand Throw	5.17 ± 1.84	5.14 ± 1.69	0.183	0.855	0.013
Underhand Throw	4.40 ± 2.04^a^	3.78 ± 1.94^a^	4.323	<0.001	0.310
Catch	3.52 ± 1.73^a^	3.28 ± 1.56^a^	2.068	0.039	0.148
One-Hand Strike	4.11 ± 1.73	4.17 ± 1.73	−0.676	0.499	−0.048
Forehand Striking	3.49 ± 1.68	3.34 ± 1.67	1.355	0.176	0.097
Two-Handed Strike	3.67 ± 1.64^a^	2.93 ± 1.44^a^	5.687	<0.001	0.408
kick	5.26 ± 1.95^a^	4.66 ± 1.68^a^	5.020	<0.001	0.360

a The *t*-test refers to a two-tailed independent sample test comparing boys and girls, Statistical significance is denoted by p < 0.05.

In contrast, the differences between boys and girls in other ball skills, such as Overhand Throw, One-Hand Strike, and Forehand Strike, were not statistically significant (p > 0.05). This suggests that these particular skills might be less influenced by gender-related factors and more by individual practice and ability.

## 4 Discussion

This study revealed that boys outperformed girls in overall ball skills, specifically in underhand throwing, catching, two-handed striking, and kicking. For locomotor skills among preschool children in Jinhua, China, there were no significant overall gender differences, but boys excelled in running-related tasks, while girls performed better in skipping-related tasks. These findings highlight distinct gender-specific motor skill proficiencies and suggest a complex interaction of genetic, environmental, and cultural factors[21,29].

### 4.1 Gender differences in height, weight, and BMI

Significant gender differences were observed in height and weight, with boys being generally taller and heavier than girls, although there were no significant differences in BMI (Table 1). Similar findings were reported by Leskinen et al. [30]in Finland, where boys were also taller and heavier, with no difference in BMI. However, other studies, such as Li et al. [31], found no consistent gender differences in height and weight among children aged 3–5.5 years. Additionally, some studies have reported no gender differences in height, weight, and BMI among children aged 4–6 years [15], possibly due to combining different age groups. The physical differences observed in our study may contribute to the variations in motor skill performance, as greater height and weight can influence strength and coordination. These physical disparities underscore the importance of considering body composition when assessing motor skills.

### 4.2 Gender differences in locomotor skills

Locomotor skills are crucial for predicting physical activity behaviors and have been associated with adherence to health recommendations regarding screen time and sleep [32]. Most studies have found no gender differences in locomotor skills among young children[15,[33], [34], [35]]. However, the complexity of gender differences in locomotor skills is highlighted by various studies presenting differing results. For instance, one study found that boys excel in running and galloping [17], while another reported that boys generally have higher overall locomotor skills compared to girls [36]. Conversely, other research indicated that girls surpass boys in locomotor skills [18]. Our team's systematic review found that gender differences in locomotor skill proficiency were nearly statistically significant, trending in favor of girls but not conclusively. (SMD = −0.07, 95 % CI -0.15 to 0.01, p = 0.09) [7]. These mixed results suggest that gender differences in locomotor skills require further investigation.

While our study found no overall gender differences in locomotor skills, a deeper analysis revealed that boys excelled in running, whereas girls performed better in skipping. These results reflect the complex findings of various studies and underscore the importance of examining specific locomotor skills individually rather than making broad comparisons.

The potential causes of these gender-specific differences in locomotor skills can be due to both evolutionary and social factors. From an evolutionary perspective, historical roles in hunter-gatherer societies might explain these differences [37]. Men often engaged in long-distance running to hunt and chase prey [38], while women primarily focused on collecting fruits, requiring abilities related to jumping and reaching. This evolutionary background might have contributed to the development of gender-specific motor skills, with men evolving to excel in running and women in jumping-related tasks.

In addition to evolutionary reasons, social and cultural factors play a crucial role. Bandura's social learning theory suggests that children learn behaviors by observing and emulating role models, such as family, peers, and teachers [39]. These role models help children learn gender roles and engage in activities that conform to these norms [40], often positively reinforced. For instance, boys are more likely to be encouraged to participate in running-related activities, while girls might be directed towards dance and gymnastics, which involve more skipping and jumping [41]. In China, cultural norms further reinforce these differences, as seen in activities like square dancing predominantly performed by women, which emphasizes coordination and rhythm skills related to skipping and jumping. Therefore, it is crucial to consider specific locomotor skills separately when assessing gender differences in motor skills. Understanding these distinctions can help in developing more targeted and effective interventions that satisfy to the unique needs of both boys and girls, promoting balanced physical development.

### 4.3 Gender differences in ball skills

Ball skills were positively associated with moderate to vigorous physical activity (MVPA) in preschoolers, However, when moderate to vigorous physical activity (MVPA) was considered as an exposure, it was not associated with locomotor skills [8].Current research generally supports that boys tend to have better ball skills than girls, a difference that becomes more pronounced with age [7]. Our study concentrated on preschool-aged children, a critical period during which discernible gender differences in ball skills emerge. The findings from our survey corroborate those of prior studies, albeit with certain variations.

Young RW [42] specifically studied human throwing and striking actions, concluding that these skills enabled ancient males to hunt more effectively, defend their territories, and attract females, thus increasing reproductive success. These actions, involving significant speed and force, are deeply rooted in neuromuscular coordination and less influenced by later learning. Boys generally prefer ball games more than girls, likely due to evolutionary advantages reinforced through learning environments, providing more opportunities to practice and develop ball skills. Our study confirms that gender differences in ball skills are evident by age 6, suggesting the need for early interventions to prevent disparities in sports participation and ensure fairness.

Young's research emphasizes the evolutionary significance of throwing and striking actions, which likely contributed to ancient males' hunting efficiency, territory defense, and mate attraction, reinforcing reproductive success [42]. Boys tend to prefer ball games, possibly due to such evolutionary predispositions, which are further shaped by environmental factors and practice opportunities.

Our study, along with Konrad et al. [43], supports the need for early and personalized physical education interventions. Particularly, it is crucial to provide tailored support for children with lower motor competencies, focusing on enhancing their learning and development in sports activities. This approach not only acknowledges the innate differences shaped by evolution but also leverages the potential for improvement through targeted educational strategies.

### 4.4 Limitations and future directions

Our research has several limitations that should be considered when interpreting the results. First, it represents gender differences in gross motor skills and does not account for all aspects of motor abilities, such as balance skills, where girls may have an advantage [31]. Second, the study was conducted in seven kindergartens in Jinhua, China, limiting its generalizability to other regions. This regional focus may not reflect broader patterns applicable to different cultural or geographical contexts. Third, as a cross-sectional study, it only provides a snapshot of associations at one point in time, and longitudinal studies are needed to understand how these differences evolve over time. Fourth, the use of subjective measures for assessing physical activity and motor skills might introduce biases, and more objective measures could provide more accurate data. Additionally, environmental and socio-cultural factors, such as parental attitudes and socio-economic status, were not fully accounted for. Fifth, the scope was limited to specific motor skills, and potential confounding variables, such as nutrition and sleep quality, were not controlled for. Addressing these limitations in future research could provide a more comprehensive understanding of gender differences in motor skill development.

Future research should explore the underlying causes of gender differences in motor skills, including genetic, environmental, and cultural factors. Longitudinal studies tracking motor skill development over time could provide insights into how these differences evolve and inform strategies for early intervention. Additionally, research should consider the role of parental and educational influences in shaping children's motor skill development, potentially offering new ways for targeted support. By recognizing these limitations and pursuing future research directions, we can develop more effective, evidence-based interventions to support the physical development of children across diverse contexts.

## 5 Conclusions

This study examined gender differences in gross motor skills among preschool children in Jinhua, China, revealing distinct proficiencies influenced by both evolutionary and cultural contexts. Boys outperformed girls in specific ball skills, such as underhand throwing, two-handed striking, and kicking. Within the locomotor skills category, boys excelled in running-related tasks, while girls performed better in skipping-related tasks. These results support the categorization of TGMD-3 locomotor skills into running-related and jumping-related tasks.The observed gender-specific differences may be attributed to cultural norms and evolutionary influences that shape motor skill development. Future research should adopt longitudinal designs and objective measures to investigate these differences over time. Additionally, considering environmental and socio-cultural factors will provide a comprehensive understanding of these gender disparities. Addressing these aspects can inform the development of gender-sensitive physical education programs, promoting balanced motor skill development for all children.

## Human ethics approval

This study was reviewed and approved by the Institutional Ethical Committee of Zhejiang Normal University with the approval number: ZSDR2019013, dated November 16, 2020. It was conducted in accordance with the Declaration of Helsinki, as well as local legislation and institutional requirements. Written informed consent for participation in this study was provided by the participants' legal guardians.

## CRediT authorship contribution statement

**Biqing Chen:** Writing – original draft, Project administration, Investigation, Funding acquisition, Conceptualization. **Yubo Liu:** Resources, Methodology, Investigation, Formal analysis, Data curation. **Jingwei Tang:** Supervision, Software, Resources, Methodology, Formal analysis, Data curation. **Jing Wang:** Visualization, Validation, Investigation, Formal analysis, Data curation. **Feng Hong:** Software, Resources, Project administration, Funding acquisition, Formal analysis. **Weibing Ye:** Writing – review & editing, Validation, Supervision, Software, Formal analysis, Conceptualization.

## Data availability

The following information was supplied regarding data availability:

The raw measurements are available in the Supplemental Files.

## Funding

This research was funded by the Jinhua City Physical Health Integration Service Procurement Project (grant number: TY2024-FW195), Zhejiang Province, China.

## Declaration of competing interest

The authors declare that they have no known competing financial interests or personal relationships that could have appeared to influence the work reported in this paper.

## Footnotes

1 * Statistical significance between boys and girls is indicated (p < 0.05).Overhand: Overhand Throwing; Underhand: Underhand Throwing; Catch: Two-Hand Catching; One Hand: One-Hand Stationary Striking; Forehand: Forehand Striking of the Self-Bounced Ball; Two Handed: Two-Hand Striking a Stationary Ball; Kick: Kicking a Stationary Ball.
